# Bacterial and fungal gut communities of *Agrilus mali* at different developmental stages and fed different diets

**DOI:** 10.1038/s41598-018-34127-x

**Published:** 2018-10-23

**Authors:** Zhengqing Zhang, Shuo Jiao, Xiaohui Li, Menglou Li

**Affiliations:** 1 Laboratory of Forestry Pests Biological Control, College of Forestry, Northwest A&F University, Yangling, Shaanxi 712100 China; 2 College of Urban and Environmental Sciences, Peking University, Beijing, 100871 China

**Keywords:** Bacterial development, Microbiome

## Abstract

*Agrilus mali* (Coleoptera: Buprestidae) is an invasive wood borer pest that has caused considerable damage to the Xinjiang wild fruit forest. In this study, we investigated the bacterial and fungal intestinal microbial communities of *A*. *mali* during different developmental stages, including larvae, pupae and newly eclosed adults or fed different diets (leaves of *Malus halliana* and *Malus pumila*) using Illumina MiSeq high-throughput sequencing technology. The results showed that microbial alpha diversity first increased and then decreased during the developmental stages, with the most dominant bacteria and fungi exhibiting the dynamic patterns “Decrease”, “Increase” and “Fluctuation”. With respect to the different diets, the bacterial communities were similar between the newly eclosed adults and adults fed *M*. *pumila* leaves, while the structure of the fungal communities showed great differences between newly eclosed adults and adults fed different diets. Through a co-correlation network analysis, we observed complex microbial interactions among bacterial and fungal taxa that were associated with potential diverse functions and intricate biological processes in the intestinal microbiota of *A*. *mali*. Overall, the results of this study demonstrated that the invasive insect *A*. *mali* harbours diverse, dynamic, and presumably multifunctional microbial communities, an understanding of which could improve our ability to develop more effective management approaches to control *A*. *mali*.

## Introduction

*Agrilus mali* (Coleoptera: Buprestidae) is an invasive wood borer that is listed as a quarantine pest in China^1^, where it primarily attacks *Malus pumila* Mill, *Crataegi cuneatae*, *Malus spectabilis*, *Prunus persica*, *Prunus armeniaca* and other economically important fruit trees^2^. *A*. *mali* was first imported from Shandong Province to the Ili Kazakh autonomous prefecture in 1993, resulting in great financial losses from damage to economically important apple trees^3^. In particular, *A*. *mali* has caused extensive damage to the Xinjiang wild fruit forest tree *Malus siversii*, which is an endangered key and priority protected species in China^4^. During its long larval stage, *A*. *mali* larvae form crooked galleries in the phloem and cambium of trees by tunnelling under the bark. Two larvae can lead to the death of a 4-cm diameter tree branch, while twenty larvae can cause the death a tree with a 15-cm diameter^1^. In contrast, although *A*. *mali* adults feed on the leaves of host plants, they have a small appetite and often do not cause very severe damage. *A*. *mali* females prefer to lay eggs on tree trunks, in bark crevices and on buds on the sunny side of trees, with one female being able to oviposit 60–70 eggs over its lifespan. In recent years, the area damaged by *A*. *mali* in the wild fruit forest of Tianshan Mountain has rapidly increased from the original 33 hm^2^ to 4,866 hm^2^, with the affected area accounting for more than half of the total wild fruit forest^4^.

Insect guts harbour diverse microorganisms that have integral roles in organismal functions, including regulating the metabolism of hosts; promoting efficient digestion to allow the maximum amount of energy to be extracted from ingested foods; aiding in the detoxification of harmful compounds; developing and maintaining the immune system of insects; and protecting hosts from potentially harmful microbes^5,6^. Associations between microorganisms and insect hosts are widespread in nature and result from the co-evolution between the microbes and hosts to generate obligate symbioses^7,8^. Studies on caterpillars of the cabbage white butterfly revealed that their gut microbial community is dominated by common environmental taxa^9^. Additionally, the gut bacterial diversity of diverse termites and cockroaches showed that host phylogeny is an important factor that determines gut microbial composition^10^. Hosting bacteria can promote specific nutritional complementation for organisms living on a markedly imbalanced diet^11,12^. Insect gut microorganisms are recognized to originate from the environment and diet^5^. For example, the acquisition of nutrition-providing bacteria in mosquito larvae is likely dependent on the presence of a particular species present in the larval habitat or the ingestion of specific bacterial species by larvae throughout their development^13^. In addition, host ecological niches and feeding habits can influence the host microbial community and have important roles in shaping the gut microbial community^5,6,14,15^.

The relationships between phytophagous insects and their parasitic/mutualistic microbes have long been investigated to gain an understanding their evolutionary diversification. In one instance, indigenous facultative or obligate mutualistic microbes are associated with several species of phytophagous insect families, including the plant-feeding insect families Chrysomelidae (Coleoptera), Curculionidae (Coleoptera), plant-galling Cecidomyiidae (Diptera) and all plant-feeding hemipteran families^16^. Symbiotic microorganisms in termites provide essential ecosystem functions by digesting cellulosic materials (wood, litter, and humus) to promote soil formation and nutrient cycling^10^. Bacterial endosymbionts are thought to be beneficial to insects by providing essential amino acids and vitamins, recycling carbon sources and defending against enemies^17^. Although fungal mutualisms are not as prevalent in phytophagous insects compared with bacterial mutualisms^18,19^, they play important roles in insect development and fitness by providing nitrogen compounds, degrading high molecular weight molecules and producing pheromones for mating and communication^20^.

Recently, the use of high-throughput next-generation sequencing technologies has provided a better and more comprehensive understanding of insect intestinal microbiotas. This technique can detect significantly higher diversity in microbial populations than traditional culture-based and conventional molecular methods^6^. Using high-throughput sequencing technologies, specific studies on bacterial and fungal communities have been performed for many insects, including termites, ants, fire bugs, fruit flies, beetles and bees, but rarely for Buprestidae insects^21–27^. One of the few studies to do so characterized gut the microbial communities of *Agrilus planipennis* via a 16S rRNA gene-based clone library profiling analysis, which suggested that the invasive insect harbours a diverse, dynamic, and presumably multifunctional microbial communities that should be viewed as multispecies complexes^28^. Another study on tissue-specific gene expression in *A*. *planipennis* demonstrated that a high number of the midgut sequences encoded chitin-binding peritrophin and trypsin domains, while sequences obtained from fat bodies encoded a high number of cytochrome P450 and protein kinase domains^29^.

In this study, investigated the bacterial and fungal communities in *A*. *mali* in detail via Illumina MiSeq sequencing. Specifically, we addressed the following objectives: 1) revealing the dynamic changes in bacterial and fungal communities over the course of the developmental stages of *A*. *mali* from natural habitats; 2) characterizing the bacterial and fungal community diversity in adult *A*. *mali* fed two different diets; and 3) exploring the distribution and assembly of the core *A*. *mali* gut microbiota.

## Results

### Distribution of taxa and phylotypes

After the quality filtering and the removal of chimeric sequences, the entire *A*. *mali* gut microbiota sequencing dataset included 416,877 high-quality bacterial sequences (bacterial V3-V4 rRNA gene region) and 117,145 high-quality fungal sequences (fungal ITS2 region) for five groups, including wild larvae (Lar), pupae (Pup) and newly eclosed adults (EcA), and the lab-reared adults fed either the leaves of M. halliana (MhaA) or M. pumila (MpuA) (Table 1). The total number of bacterial and fungal OTUs identified was 20,704 and 1,969, respectively, defined at a 97% sequence similarity (Table 1). Rarefaction curves suggested that the majority of the bacterial and fungal taxa were recovered (see Fig. S1 in supplementary material). Among the bacterial OTUs, 99.02% (20,502 OTUs) were assigned to 39 phyla, 96 classes, 144 orders, 202 families, 334 genera and 216 species. Among the fungal OTUs, 89.54% (1,763 OTUs) were assigned to 5 phyla, 21 classes, 53 orders, 125 families, 255 genera and 423 species. In general, the number of bacterial OTUs was larger than that of fungi. Four bacterial phyla, including Proteobacteria, Actinobacteria, Firmicutes and Bacteroidetes were predominant (relative abundance >1%), and accounted for 77.42% of the total sequences. The phylum Proteobacteria, with a relative abundance of 70.68%, was represented by the classes Gammaproteobacteria (46.69%), Alphaproteobacteria (16.30%) and Betaproteobacteria (6.20%). In particular, Pup contained large number of specific phyla, including Caldithrix, Chlamydiae, Elusimicrobia, Gemmatimonadetes, Planctomycetes, Nitrospirae and Verrucomicrobia, which were not detected or showed low relative abundances in the other samples. At the fungal phylum level, Ascomycota and Basidiomycota were the predominant phyla, with relative abundances of 73.11% and 16.63%, respectively.

**Table 1:** Microbial community alpha-diversity characteristics in the guts of *Agrilus mali* at different developmental stages and fed different diets.

Sample ID	Tag number	Alpha diversity indices				
		OTU richness	Shannon-Wiener	Chao 1	ACE	Coverage
**Bacteria**						
Lar	86,187	6,608	5.01	15,366.31	23,082.41	98.42%
Pup	86,176	10,861	5.31	46,041.04	103,172.74	98.28%
EcA	72,284	1,198	1.33	2,408.08	3,583.91	90.28%
MhaA	83,728	2,140	2.14	4,987.25	8,717.10	95.46%
M.puA	88,502	2,542	2.19	5,784.24	9,240.62	99.08%
**Fungus**						
Lar	6,862	433	3.86	793.00	1354.11	91.82%
Pup	8,367	650	4.24	1,344.11	2071.72	95.32%
EcA	91,179	541	1.74	976.76	1321.88	95.79%
MhaA	3,216	447	4.36	985.33	1693.34	96.72%
MpuA	7,521	642	4.29	1,320.86	1957.63	99.72%

### Microbial alpha and beta diversity in the guts of *A. mali*

**Figure 1:**
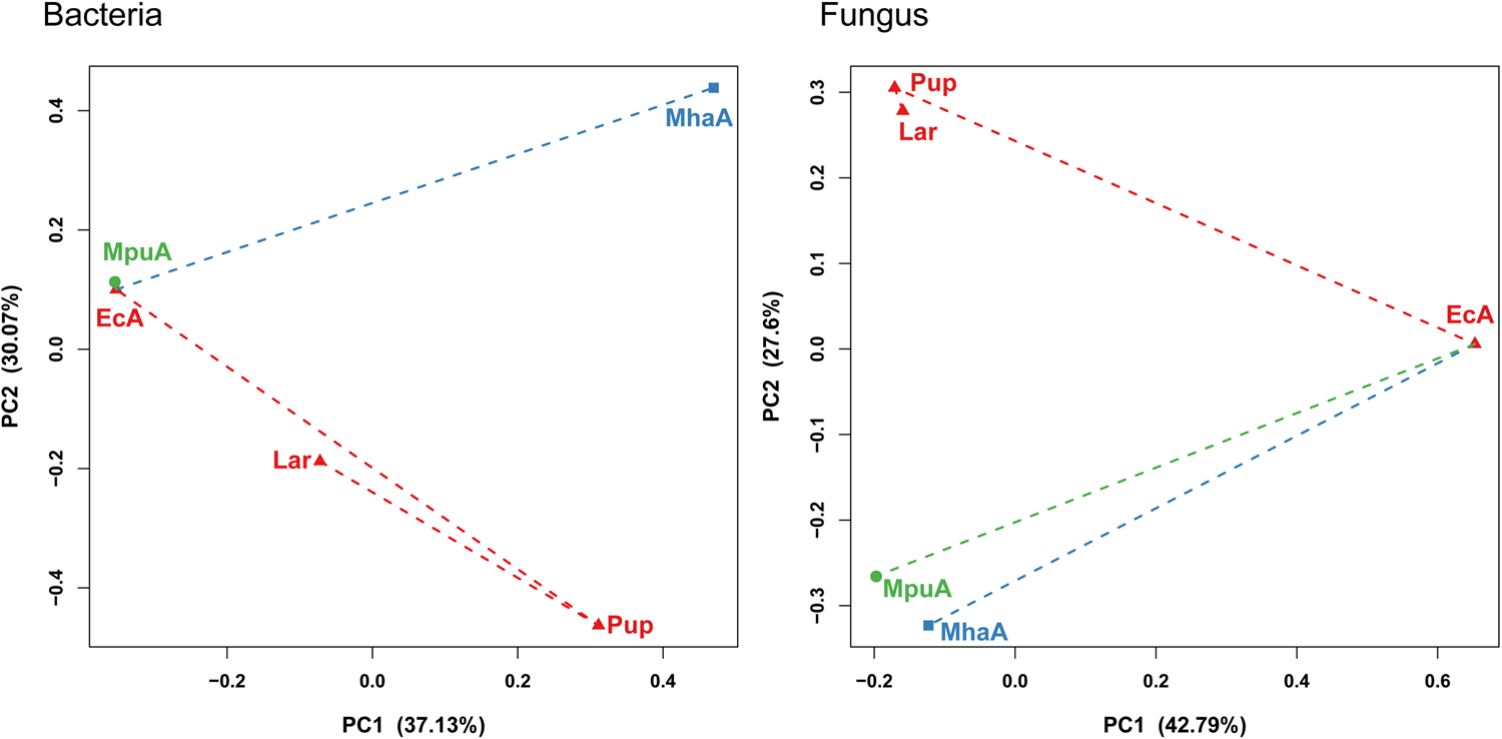
Microbial diversity patterns for *A*. *mali* gut microbiotas. Alpha-diversity in bacterial (**A**,**C**) and fungal (**B**,**D**) gut communities of *A*. *mali* gut. Beta-diversity in bacterial (**E**) and fungal (**F**) gut communities estimated via principal coordinate analysis (PCoA) based on Bray-Curtis distance.

The microbial alpha diversity indices, including the OTU richness (i.e., richness) and the Shannon-Wiener index (i.e., Shannon) were estimated in the guts of *A*. *mali* during three developmental stages and for insects fed two different diets (Fig. 1A–D). Across different developmental stages, the microbial alpha diversity showed a trend of first increasing and then decreasing. The Pup group showed highest OTU richness and Shannon-Wiener index for both bacterial and fungal communities. For the bacterial communities, the alpha diversity indices were lowest in the EcA group, while for the fungal communities, the OTU richness and Shannon-Wiener index was lowest in the Lar and EcA groups, respectively. Considering the effect of different diets, the MpuA group (adults fed *M*. *pumila* leaves) showed a higher bacterial Shannon-Wiener index than that of the MhaA group (adults fed *M*. *halliana* leaves), and the bacterial OTU richness differed little between the two gut microbiotas. In contrast, the fungal OTU richness was higher in the MpuA group than that observed in the MhaA group, whereas the Shannon-Wiener index differed little between the two gut microbiotas.

Principal coordinate analysis (PCoA) based on the Bray-Curtis distance (Fig. 1E,F) showed different results between the bacterial and fungal communities of the gut microbiota in *A*. *mali*. For the bacterial community, the microbiota varied in succession across the developmental stages, where the MpuA and EcA groups clustered together away from the MhaA group. For the fungal community during the different *A*. *mali* development stages, the Pup and Lar groups clustered together and were separated from the EcA group. In contrast, in the treatment of *A*. *mali* with different diets, the MpuA and MhaA groups clustered together and were separated from the EcA group.

The observed differences in beta-diversity resulted from the variations in community structure of the gut microbiotas (Supplementary Fig. S2). The relative abundance of Proteobacteria decreased along with the development stages and was much higher in the MhaA group (above 95%) than in the MpuA and EcA groups. The relative abundance of Ascomycota increased along with the development stages, reaching 98% in the EcA group. Greater differences were observed at the class level among the gut microbiotas (Supplementary Fig. S3). Gammaproteobacteria represented the predominant taxa in the phylum Proteobacteria, showing a consistent trend with that of Proteobacteria, while the fungal class Eurotiomycetes and phylum Ascomycota exhibited similar trends. Comparatively, the relative abundance of Alphaproteobacteria was highest in EcA group, and Betaproteobacteria were most abundant in the Pup group. The relative abundance of Actinobacteria was lowest in the MhaA group compared with that observed in the other microbiotas. Additionally, the relative abundances of Bacilli were 5.32% and 6.27% in the Lar and Pup groups, respectively but were very low in the EcA, MhaA and MpuA groups. In contrast, the fungal taxa Dothideomycetes and Sordariomycetes were the most abundant in the EcA group compared with those observed in the other samples In addition, the fungal taxa Wallemiomycetes, Tremellomycetes, Saccharomycetes and Agaricomycetes contributed a substantial fraction of reads in the Lar and Pup groups, but were rare in the EcA group. Moreover, the bacterial community structure in the EcA group was more similar to that of the MpuA group than the MhaA group, and the fungal community structure in the MhaA and MpuA groups were similar and different from that observed in the EcA group.

### Gut microbial dynamic patterns during the development of *A. mali*

**Figure 2:**
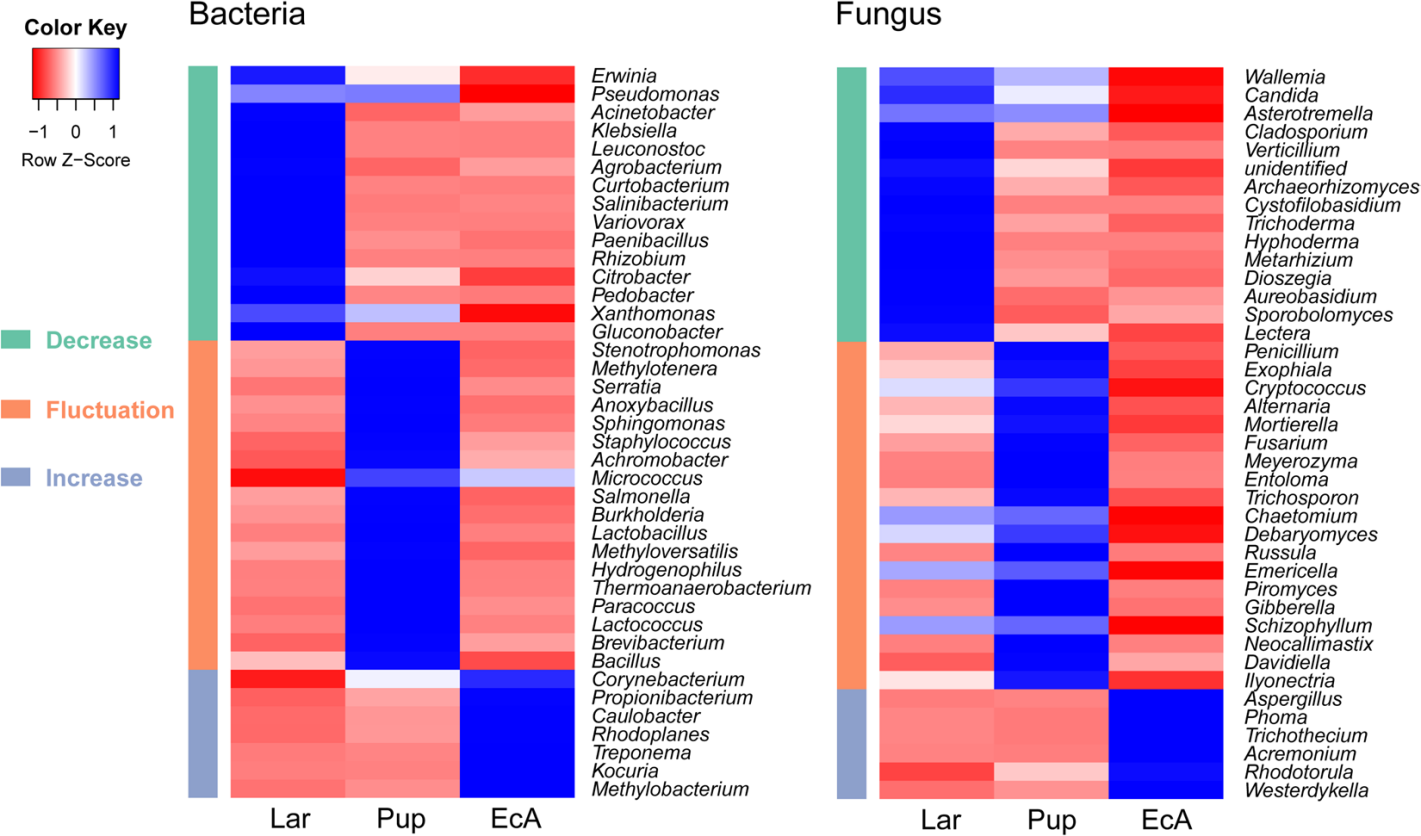
Dynamic patterns of bacterial and fungal communities during the development of *A*. *mali* gut microbiotas, as determined using cluster analysis. The 40 genera with the highest relative abundances were selected and clustered into three groups on the basis of similar profiles (displayed as heatmaps). Each row in the heatmap has been standardized to have a mean of zero and a standard deviation of one. The intensity of the colour in the heatmap is proportional to the standardized relative abundances of the taxa.

To explore the dynamic patterns of gut microbial communities during the development of *A*. *mali*, a cluster analysis was performed for the datasets obtained from the three developmental stages. As we focused on the abundant taxa, the 40 genera with the highest relative abundances were selected for this analysis. Clustering of these genera into three groups indicated that bacterial and fungal taxa showed similar dynamic patterns, including “Decrease”, “Increase” and “Fluctuation” patterns (Fig. 2). “Decrease” indicates microbes that were high in abundance in larvae; “Increase” indicates microbes that increased in abundance from larvae and pupae to eclosing adults; and “Fluctuate” represents microbes that were high in abundance in pupae. During the development of *A*. *mali*, the abundances of the bacterial taxa *Erwinia*, *Pseudomonas*, *Acinetobacter*, *Klebsiella*, *Leuconostoc* and *Agrobacterium* decreased continuously; *Stenotrophomonas*, *Methylotenera*, *Serratia*, *Anoxybacillus*, *Sphingomonas* and *Staphylococcus* first increased and then decreased; and *Corynebacterium*, *Propionibacterium*, *Caulobacter*, *Rhodoplanes*, *Treponema*, and *Kocuria* increased continuously. For the fungal taxa, *Wallemia*, *Candida*, *Asterotremella*, *Cladosporium* and *Verticillium* decreased continuously; *Penicillium*, *Exophiala*, *Cryptococcus*, *Alternaria*, *Mortierella* and *Fusarium* first increased and then decreased; and *Aspergillus*, *Phoma*, *Trichothecium*, *Acremonium*, *Rhodotorula* and *Westerdykella* increased continuously. In general, our results may indicate the presence of microbial community succession in the gut microbiota during the development of *A*. *mali*.

### The effect of different diets on the assembly of the *A. mali* gut microbiota

**Figure 3:**
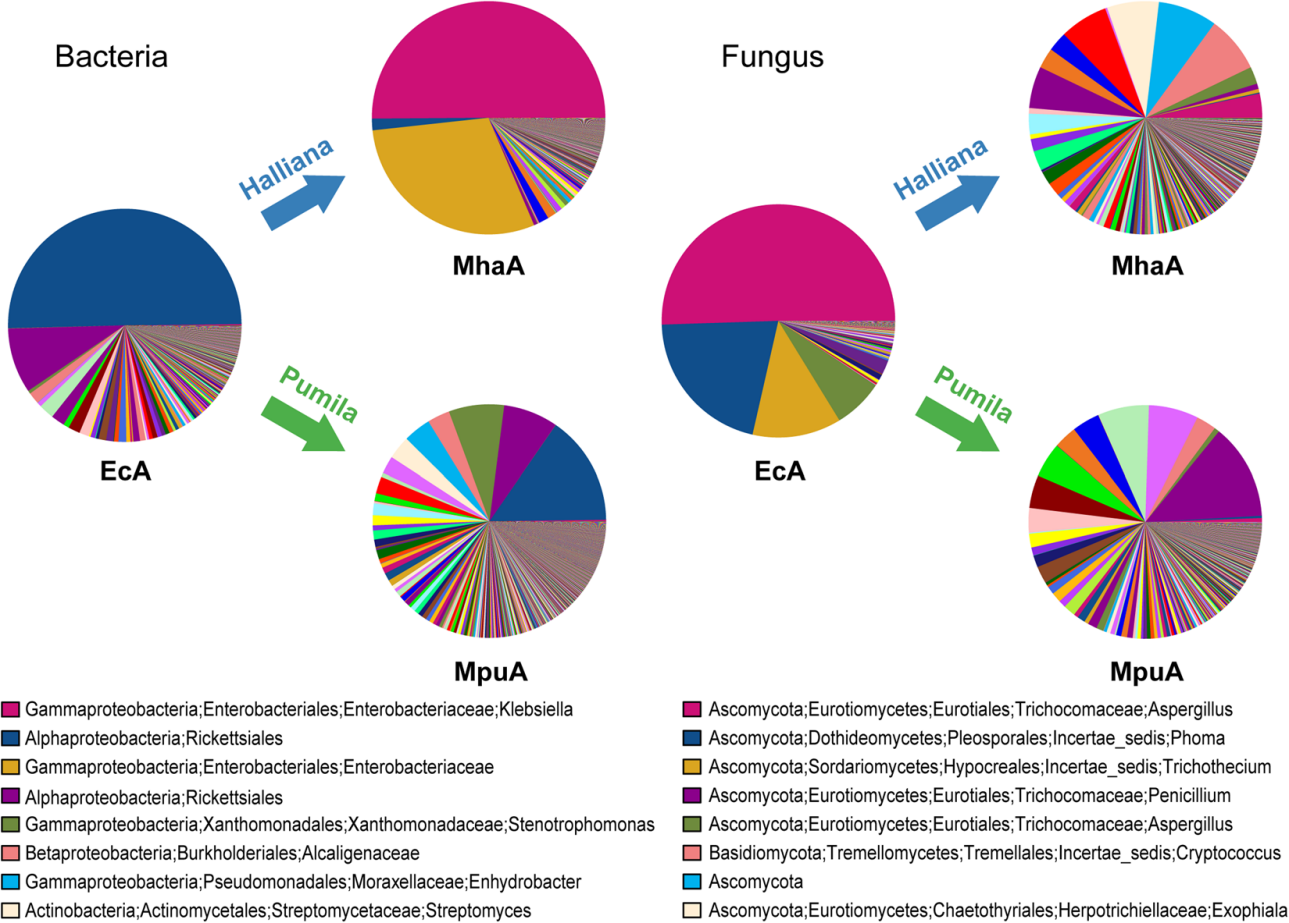
Pie charts of the bacterial and fungal OTU abundances in gut microbial communities of *A*. *mali* fed different diets of Halliana and Pumila leaves. The taxonomic information of the OTUs with the six highest relative abundances are displayed in the legends.

The effect of different diets on the assembly of the *A*. *mali* gut microbiota was assessed based on the observed OTUs (Fig. 3). In bacterial communities, the predominant OTUs in the EcA groups were also dominant in the MpuA group, which primarily belonged to the taxon Rickettsiales, whereas the dominant OTUs in the MhaA group primarily belonged to the taxa *Klebsiella* and Enterobacteriaceae, different from that observed for EcA. Some OTUs became abundant in the MpuA group compared with the EcA group, which were assigned to the genera *Stenotrophomonas*, *Enhydrobacter* and *Streptomyces*. For fungal communities, the predominant OTUs in the EcA group were not abundant in the MpuA and MhaA groups. In addition, the community compositions between the MpuA and MhaA groups were similar, but the relative abundances of the OTUs were differed greatly. For example, the taxa *Cryptococcus podzolicus*, *Exophiala salmonis*, *Aspergillus cibarius*, *Stachybotrys microspora*, *Fusarium* and *Archaeorhizomyces* were dominant in the MhaA group, while the taxa *Penicillium citrinum*, *Penicillium menonorum*, *Candida*, *Russula*, *Penicillium commune* and *Trichosporon asahii* were abundant in the MpuA group. Thus, these observations confirmed that the bacterial community structure in the EcA group was more similar with that of the MpuA group than the MhaA group, whereas the fungal community structures of the MhaA and MpuA groups were similar but differed from that of the EcA group.

### Co-correlation network among bacterial and fungal taxa

**Figure 4:**
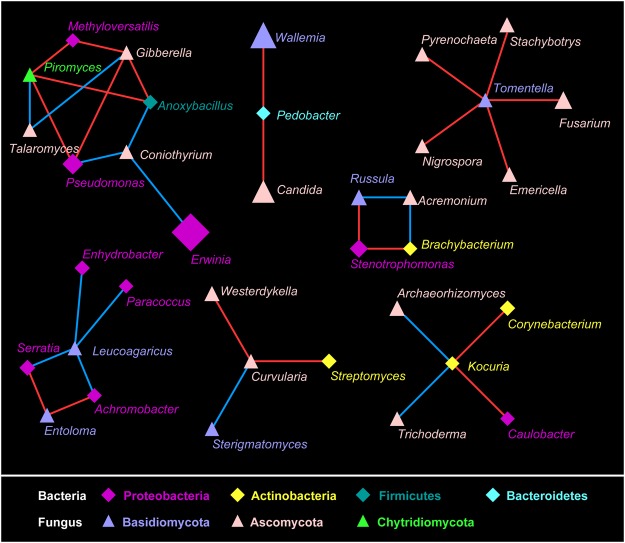
Co-correlation network of microbial bacteria and fungus genera in the *A*. *mali* gut microbiota. A connection indicates for Spearman’s correlation with a coefficient >0.6 (positive correlation, red edges) or <−0.6 (negative correlation, blue edges) and a significant (*P* < 0.01) correlation. The size of each node is proportional to the relative abundance. Diamonds indicate bacteria, and triangles indicate fungus. The nodes were coloured by phylum.

The correlation network was generated to unravel the microbial co-occurrence patterns among the bacterial and fungal taxa in the gut microbiota of *A*. *mali* (Fig. 4). In general, the network consisted of 36 nodes and 35 edges (with 21 positive edges and 14 negative edges). These nodes were distributed into four bacterial phyla, including Proteobacteria, Actinobacteria, Firmicutes and Bacteroidetes, and three fungal phyla, including Basidiomycota, Ascomycota and Chytridiomycota. We observed complex interactions among bacterial and fungal taxa. For example, *Coniothyrium* was negatively correlated with *Erwinia*, *Pseudomonas* and *Anoxybacillus*; *Gibberella* was positively correlated with *Methyloversatilis* and Pseudomonas and *Anoxybacillus*; *Tomentella* was linked by positive edges with *Fusarium*, *Emericella*, *Stachybotrys*, *Nigrospora* and *Pyrenochaeta*; and *Leucoagaricus* was linked by positive edges with *Serratia*, *Paracoccus* and *Enhydrobacter*.

### Core microbiota taxa of *A. mali*

**Figure 5:**
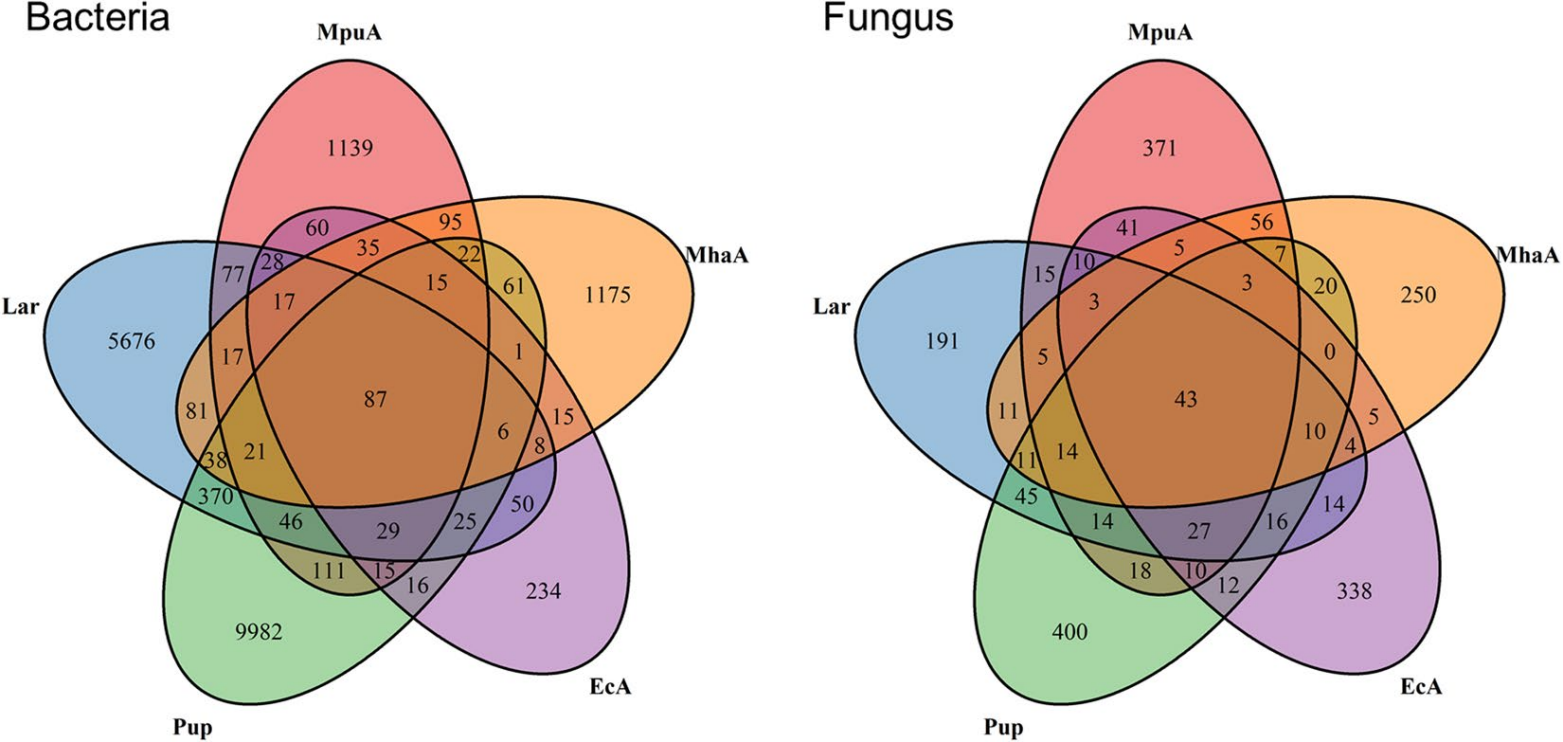
Venn diagram of the bacterial and fungal OTUs in different gut microbiotas of *A*. *mali*.

The persistent microbial taxa in the gut microbiota of *A*. *mali* were identified based on the OTUs present in all five samples (Fig. 5). A total of 87 and 43 OTUs were identified and defined as core taxa in the bacterial and fungal communities, respectively, accounting for low proportions of the total OTUs (means = 3.37% and 8.16% for bacteria and fungi, respectively). In contrast, these core taxa accounted for a substantial fraction of the reads (means = 44.43% and 59.56% for bacteria and fungi, respectively; Supplementary Fig. S4). The bacterial core taxa were primarily assigned to *Klebsiella*, *Stenotrophomonas*, *Serratia*, *Enhydrobacter*, *Achromobacter*, *Corynebacterium*, *Micrococcus* and *Acinetobacter*; and the fungal core taxa primarily belonged to *Aspergillus*, *Wallemia*, *Phoma*, *Candida*, *Penicillium*, *Cryptococcus*, *Cladosporium* and *Fusarium* (Supplementary Table S1 and S2).

## Discussion

Insects harbour diverse gut microorganisms that participate in many different activities, including the degradation of lignocellulose; the production of nutrients, vitamins and components of cohesion pheromones; nitrogen fixation and utilization of nitrogenous waste products; protection against parasites; the promotion of changes in body colouration; and sterol synthesis^15,28–31^. Although accumulating studies have described the microbial diversity in the insect gut, especially in Coleoptera insects^15,28,30,32–37^, to date, no report had described changes in *A*. *mali* gut microbial communities during successive life stages and in response to different diets. In the present study, we provide a novel description of the intestinal microbiota related to this apple buprestid via Illumina MiSeq high-throughput sequencing of the bacterial 16S rDNA gene and fungal ITS gene regions.

### Microbiota richness and diversity of *A. mali* Gut

In general, the annotation results revealed that *A*. *mali* contained a larger number of bacterial than fungal taxa. Similar results have been reported in members of the families Passalidae and Elateridae, while members of the family Cerambycidae have shown a high diversity of fungi and a moderate diversity of bacteria^38^. A previous study demonstrated that the coverage of bacterial gut communities is often sufficient between individuals, while fungal diversity is generally underestimated^30^. Additionally, we observed that the dominant gut microbial communities in *A*. *mali* only occupied a relatively low numbers, consistent with previous findings in pea aphids and red palm weevils^39,40^. Across the different *A*. *mali* development stages, pupae showed the highest microbial diversity in gut microbial communities, a result that was also observed for the guts of the emerald ash borer^28^. Insect gut microorganisms are recognized to originate from the environment and diet of a given species^5^. The high gut microbial diversity observed in *A*. *mali* pupae may have resulted from the vast microbial influx occurring via a specific nutritional complementation from a markedly imbalanced diet^11–13^. Another explanation is that the sequencing effort might cause a greater number of transient, non-specific bacteria to be present in pupae, since the pupae lose much of their core gut bacteria during the process of pupation.

Compared with the newly eclosed adults, *A*. *mali* adults fed different diets showed increased diversity in gut microbiota. We hypothesized that feeding might stimulate the growth of microbiota and provide new taxa from the diet to restore the community richness in the insect gut^41^. However, our results showed that there were substantially more unique OTUs that shared OTUs between any two life stages, which seemed odd given the life history of this insect and might indicate contamination of our samples. Thus, more stringent procedures for controlling contamination will be conducted in our future research.

The comprehensive community analysis across the developmental stages of *A*. *mali* and in insects fed different diets revealed that four bacterial phyla, including Proteobacteria, Actinobacteria, Firmicutes and Bacteroidetes, and two fungal phyla, including Ascomycota and Basidiomycota, were predominant. This result is supported by a previous study is similar to that of a previous study showing that Proteobacteria and Firmicutes were the predominant bacterial phyla in 81 insect gut samples^6^. Essentially, species belonging to all major bacterial taxa have been isolated from bark beetles, including Alphaproteobacteria, Bacteriodetes, Firmicutes, Betaproteobacteria, Gammaproteobacteria and Actinobacteria^42^. The dominant fungal phyla observed in *A*. *mali* is consistent with previous observations of a high diversity of Basidiomycota and Ascomycota isolates from Cerambycidae, Passalidae, Elateridae and the Hemiptera insect genus *Dactylopius*^20,38^. The assembly of these communities in insect alimentary tract microbiota could be determined by the physiological and biochemical conditions in this environment, such as gut morphology, pH and oxygen availability^5,43^.

### Gut microbiota variation during different developmental stages

According to the PCoA analysis, the gut microbiota varied in succession across the *A*. *mali* developmental stages, with pupae being clearly separated from larvae and newly eclosed adults. Compared with larvae and adults, the pupal gut undergoes a decrease in metabolic activity and undergoes morphological changes during the insect metamorphosis that may influence the associated microbial communities^36^.

A complex microbial consortium is considered fundamental for normal survival and complete development of insects from larvae to adults^13^. During the development of *A*. *mali*, the dominant gut bacterial and fungal communities primarily exhibited three dynamic patterns, including “Decrease”, “Increase” and “Fluctuation”. For the bacterial community, cellulolytic bacteria, including members of the genera *Erwinia*, *Pseudomonas*, *Acinetobacter*, *Klebsiella*, *Leuconostoc* and *Agrobacterium* were more abundant in xylophagous *A*. *mali* larvae, which feed on phloem and cambium^28^. Interestingly, *Klebsiella* has been used in cellulose degradation and fermentation processes^44^. In addition, *Klebsiella* sp. are recognized as recurrent diazotrophs that contribute to beetle nitrogen requirements and sometimes are closely related to pathogenic bacteria obtained from the environment^32,33^. *Pseudomonas* has been isolated in most Coleoptera insects due to its encoded cellulolytic enzyme capacity, which may be involved in terpene transformation of plant resin compounds^36,45,46^. For the fungal community, a continuous decrease in the fungal taxa *Wallemia*, *Candida*, *Asterotremella*, *Cladosporium* and *Verticillium* were also observed to be abundant in larvae. Ascomycetous yeast of the genus *Candida* and basidiomycetous yeast of the genus *Asterotremella* were shown to produce xylanase and carboxymethyl cellulose in insects and their natural habit^47^. *Wallemia* and *Cladosporium* are very common environmental fungal genera that associate with many plants as commensals and pathogens^48^. These microbial groups are widespread, ecologically diverse, and functionally redundant in organic matter decomposition and lignocellulose digestion and may be transiently acquired by insects from feeding materials and the environment^10^.

Some other bacteria and fungi showed “Increase” patterns during developmental stages that were relatively abundant in newly eclosed adults. The abundances of the bacterial genera *Propionibacterium* and *Kocuria* were extremely high in newly eclosed adults, which has been observed in the guts of other insect, e.g., the bark beetle *Dendroctonus rhizophagus*, *A*. *planipennis* and the hornworm *Manduca sexta*^32,36^. Interestingly, *Propionibacterium*, which was identified in *A*. *mali* adults, was reported to cause skin acne in humans^15^. For the fungal community, genera in the classes Dothideomycetes, Sordariomycetes, Eurotiomycetes were primarily detected in newly eclosed adults. These groups are involved in organic matter decomposition and are saprobes or endophytes that associate with plants, arthropods, and mammals^10^. The observed “Fluctuation” pattern of microbiota included the bacterial genera *Stenotrophomonas*, *Serratia*, *Sphingomonas* and *Staphylococcus* and the fungal genera *Penicillium*, *Cryptococcus* and *Fusarium*. *Stenotrophomonas* has been described to protect hosts against an entomopathogenic fungus in the fly *Stomoxys calcitrans*. *Sphingomonas* is known as an endosymbiont of ticks^14,15^. *Serratia* is often isolated from the guts of insects in the wood-feeding Coleoptera order, possessing potential characteristics related to the degradation of lignocellulose and xylan and for fermentative metabolism^34,38^. *Staphylococcus* is commonly present in the environment and is a symbiont in the guts of many insect, including *Drosophila melanogaster*, *Culex quinquefasciatus*, *Analeptes trifasciata* and *Asobara tabida*^46,49^. *Cryptococcus* and *Penicillium*, yeast-like symbionts, were reported to be involved in uric acid (UA) catabolism via turning UA into amino acids for insects^20^. In addition, *Fusarium* was observe to be associated with amino acid salvage and the recycling of nitrogenous waste products^35^. The evolutionary innovation of holometaboly also created distinct niches for colonization by distinct microbial symbionts. Over the holometabolous life cycle of the host, variation in diet and internal physicochemical conditions could support communities functionally specialized for a particular life stage^41^.

### Gut microbiota variation with different diets

Gut microbial communities can be influenced by host diet, as they adapt to dietary changes through the induction of enzyme production and changes in community structure^30^. The bacterial communities were similar between the newly eclosed adults and adults fed *M*. *pumila* leaves, whereas great differences in fungal community structures were observed between newly eclosed adults and adults fed either diet. This result indicates that bacterial taxa inhabiting the gut of *A*. *mali* are more adapted to *M*. *pumila* leaves and that the fungal community is more easily affected by different diets.

In the bacterial community, the predominance of the order Rickettsiales in the EcA group was also observed in the MpuA group but not the MhaA group, in which *Klebsiella* and Enterobacteriaceae were dominant. In the present study, larvae were fed cambium and phloem, while adults were fed foliage, possibly indicating that the bacterial community was conserved in larvae and adults (MpuA) regardless the shift in diet after metamorphosis. Previous studies have demonstrated that dietary influences on adult gut microbiota were obscured by colonization history^33,50^. Because adult *A*. *mali* ingest large amounts of foliage, which contains potentially useful and harmful microbes associated, it makes sense for the host to efficiently control its gut microbiota and quickly eliminate invading microbes^51^. Rickettsiales species are widespread insect symbionts that have mutualistic fitness effects in their hosts and commonly cause pathogenicity or reproductive alterations^15,32^. Members of the family Enterobacteriaceae are often dominant taxa in insect guts due to their various abilities, including hydrolysing and fermenting carbohydrates, catalysing nitrogen fixation, and producing vitamins and pheromones^33^. In addition, shifts in diet may be beneficial to the colonization of Enterobacteriaceae taxa, which often showed strong adaptability.

For the fungal communities, the predominant OTUs in the EcA group were not abundant in the MpuA and MhaA groups, and the community compositions between the MpuA and MhaA groups were similar differed greatly in abundance. The feeding behaviour of *A*. *mali* adults may explain the similar fungal composition between the MpuA and MhaA groups^10^. Both groups were fed leaves of different plant species collected from the same location, and many insects derive their gut microbiota from the surrounding environment, including the phylloplane of food plants^43^.

### Complex interactions among gut microbiota in *A. mali*

Microorganisms form complex interaction webs within specific ecological niches, and understanding the interactions among microorganisms is important to explore the complexity of functional processes^52^. In the present study, we observed complex interactions among bacterial and fungal microbes. The bacterial genera *Corynebacterium* and *Kocuria* were observed to be positively correlated in the *A*. *mali* gut, two genera that were previously observed to co-occur in the bacterial gut microbiota of the bark beetle^53^. With respect to fungi in the *A*. *mali* gut, *Tomentella* was positively correlated with *Fusarium*, *Emericella*, *Stachybotrys*, *Nigrospora* and *Pyrenochaeta*, suggesting that they potentially share similar ecological niches in the intestinal microenvironment. More intricate linkages were observed between bacterial and fungal microbes. In general, there were 12 positive and 10 negative edges between taxa of these two groups. Interactions among microorganisms may be associated with specific functions possessed by gut microbiota. In the co-correlation network, *Coniothyrium*, reported as an antimicrobial fungus^54^, was negatively correlated with the bacterial genera *Erwinia*, *Anoxybacillus* and *Pseudomonas*. *Kocuria*, which was negatively correlated with the fungal genera *Archaeorhizomyces* and *Trichoderma*, has been observed to have strong antifungal activity^55^. Overall, the complexity of microbial interactions was associated with multiple functions of gut microbiota, indicating that intricate biological processes exist in intestinal microenvironment of *A*. *mali*.

### Core taxa associated with potential gut microbiota functions in *A. mali*

In the *A*. *mali* gut microbiota, we identified only a few core taxa, which accounted for a substantial fraction of the total reads. Their persistence in the gut microbiota during different development stages and with different diets suggest that they may be important for maintaining microbial diversity and intestinal health in *A*. *mali*^56^. Some of specific enzymatic degrading activities of these core bacteria are thought to have important roles in the intestinal microenvironment of *A*. *mali*, including the detoxification of plant compounds, the production of metabolites against pathogens, and plant-insect interactions. For example, *Stenotrophomonas* and some other Proteobacterial bacteria were reported to have cellulose and/or aromatics degradation capabilities^57,58^. Members of the fungal genus *Fusarium* are abundant members in the *A*. *mali* gut, similar to the *A*. *glabripennis* larval midgut and are capable of secreting numerous plant cell wall degrading enzymes, detoxification enzymes, and laccases with potential involvement in lignin degradation^35,59^. Insects tolerant microbial intruders both inside and outside their bodies, and symbiotic microbiota simultaneously supply enzymes that generate metabolites for host utilization. The presence of core microbiota in the gut indicated that there may well be core microbial functions in healthy individuals^56^. In a previous study, insects were observed to produce a large number of transcripts predicted to break down plant cell-wall carbohydrates, whereas the gut microbial community expressed numerous transcripts predicted to be involved in the assimilation and fermentation of wood sugars^35^. Moreover, the gut microbiota in *Anoplophora glabripennis* can also contribute to nitrogen availability by recycling nitrogenous waste products that are reincorporated into both essential and non-essential amino acids^60^.

## Conclusion

In this study, we comprehensively investigated the gut microbiota of *A*. *mali* during development and in adults fed different diets obtained from the same locality. During development, most dominant bacteria and fungi showed the dynamic patterns of “Decrease”, “Increase” and “Fluctuation”. The bacterial communities were similar between the newly eclosed adults and adults fed *M*. *pumila* leaves, whereas the structure of fungal communities from these two groups exhibited large differences between newly eclosed adults and adults fed with different diets. These results indicated that bacterial taxa in the *A*. *mali* gut were more adapted for *M*. *pumila* leaves, whereas the fungal community was more easily affected by different diets. In addition, we observed that complex microbial interactions may be linked to diverse functions of the gut microbiota, reflecting intricate biological processes in the intestinal microenvironment of *A*. *mali*. The results of this study could improve our ability to develop more effective management approaches in controlling *A*. *mali* in China, especially in regions that commonly suffer from outbreaks, such as Xinjiang Province. However, additional studies must still be performed, such as characterizing the specific members and roles of gut microbiota in *A*. *mali*. Thus, future research should determine the mechanisms of microbial passage and loss among the different life stages of *A*. *mali* and determine processes by which gut flora affect host plant utilization and cause host illness.

## Materials and Methods

### Sample Collection

Damaged branches of *Malus siversii* were collected from the wild fruit forest located in Yining city, Ili Kazakh autonomous prefecture, Xinjiang Province, China, on June 26, 2015. The larvae, pupae and some newly eclosed adults of *A*. *mali* were collected from the damaged branches and stored in centrifuge tubes, after which the guts were immediately dissected. The remaining newly eclosed adults, which were hatched from larvae collected from the damaged branches of *Malus siversii*, were reared with fresh leaves from different apple tree species, including *Malus halliana* Koehne and *Malus pumila* Miller, in an artificial climate chamber at 25 ± 1 °C with a 70 ± 5% relative humidity and a photoperiod cycle of 16/8 (L/D). The experimental feeding period was 20 days (from July 4 to July 24, 2015) to ensure the formation of intestinal microflora. Twenty individuals from each sample were manually selected and gut dissection was performed within 4 h.

### Gut Dissection and DNA Extraction

Twenty individuals each of the five sample groups were used for gut dissection, including the wild larvae (Lar), pupae (Pup) and newly eclosed adults (EcA), and the lab-reared adults fed either the leaves of *M*. *halliana* (MhaA) or *M*. *pumila* (MpuA). Each sample was superficially disinfected with 75% ethanol for 1–3 min and then rinsed repeatedly with sterile water. Next, the insects were transported to a horizontal clean bench, and the guts were extracted with sterile fine tip forceps under a stereoscope and placed into 1.5-ml microcentrifuge tubes. Gut dissections were performed under sterile conditions and included the removal of the hepatopancreatic gland. The guts of twenty individuals from each sample group were pooled in one tube and crushed gently with a pestle in liquid nitrogen. Then, the gut tissue was transferred into 100 μl of sterile PBS. The tissue samples were macerated with sterile polypropylene micro pestles inside 1.5 ml tubes, after which the tubes were centrifuged at low speed to pellet the macerated gut tissue. Total DNA was extracted from each sample using a General AllGen Kit (ComWin Biotech Co., Ltd., Beijing, China) according to the manufacturer’s instructions. The extracted DNA was stored at −80 °C and reserved for further use.

### PCR amplification and sequence data processing

The V3 and V4 hypervariable regions of the bacterial 16S rRNA gene were amplified using the specific barcoded primers 341 F (5′-CCTAYGGGRBGCASCAG-3′) and 806 R (5′-GGACTACNNGGGTATCTAAT-3′). The ITS2 region of the ITS rDNA was amplified using the specific barcoded primers F (5′-GCATCGATGAAGAACGCAGC-3′) and R (5′-ATATGTAGGATGAAGAACGYAGYRAA-3′) to assess fungal diversity. The PCR reaction mixtures (25 μl) contained 10 pmol of each primer, 5–10 ng DNA and 1× GeneAmp PCR Gold Buffer, 3.5 mM MgCl_2_, 0.2 mM GeneAmp dNTPs, and 0.025 U/μl AmpliTaq Gold DNA Polymerase, LD (Applied Biosystems, Foster City, CA). The PCR amplification procedure was as follows: 95 °C for 10 min, followed by 35 cycles of 95 °C for 30 s, an incubation at the appropriate annealing temperature (see below) for 30 s and 72 °C for 25 s, and a final extension step of 72 °C for 10 min. The annealing temperatures used were 52 °C and 57 °C for the bacterial V3-V4 and fungal ITS2 regions, respectively. All samples were amplified in triplicate, and no-template controls were included at all steps of the process. The PCR reaction products were detected by agarose gel electrophoresis and purified using a QIAquick Gel Extraction Kit (QIAGEN, cat#28706). The purified DNA was quantified using a QuantiFluor Fluorometer (Promega, SA3060) and equivalent amounts were subsequently mixed. Barcoded V3-V4 and ITS2 amplicons were ligated with adapters to construct sequencing libraries, which were sequenced using the paired-end method with an Illumina MiSeq (250-bp paired-end reads) with a 6 cycle index read at GENE DENOVO Co., Ltd. (Guangzhou, China).

Raw reads were removed that had contaminating adaptors, were of low quality and had polymeric sequences longer than 10 bp, had ambiguous bases, or had mismatched primers. For the V3-V4 and ITS2 paired-end reads, only clean sequences with overlaps of longer than 10 bp and with a mismatch rate of lower than 0.02 were assembled according to their overlapping sequences. The assembled reads (called tags) were used to remove redundant sequences with Mothur v.1.34.0^61^. Sequences with ≥97% similarity was assigned to the same OTU (operational taxonomic unit). The low-abundance OTUs were eliminated from the OTU table if they did not have at least 2 counts across all the samples in the experiment. Representative sequences for each OTU were assigned to taxonomic groups using the Ribosomal Database Project naïve Bayesian rRNA classifier with the Greengenes database.

### Data analysis

Alpha diversity indices, including the OTU richness, the Shannon-Wiener index, the bias-corrected Chao1 richness estimator, and the abundance-based coverage estimator (ACE) were determined using Mothur v1.34.0 and were used to analyse the microbial species diversity in each sample^62^. To identify linkages between the samples, the beta diversity among different samples was estimated based on the pairwise Bray-Curtis dissimilarity distances. PCoA was performed on the distance matrices to visualize the sample relationships. Dynamic patterns of bacterial and fungal communities during the development of the *A*. *mali* gut microbiota were determined using cluster analysis. The 40 genera with the highest relative abundances were selected and clustered into three groups-based similar profiles, which were displayed in a heatmap. A pie chart was constructed to analyse community variations in *A*. *mali* gut microbiotas resulting from different diets. Venn diagrams were generated to evaluate shared and unique bacterial and fungal OTUs to describe the similarities and differences among the different samples and treatments.

A network analysis was used to explore interactions between bacterial and fungal taxa. Bacterial and fungal genera with relative abundances above 0.05% were selected for this analysis. A Spearman’s correlation between two genera was considered significantly robust if the coefficient (ρ) was >0.6 or <-0.6, and the *P*-value was <0.01. All of the robust correlations identified from pairwise comparisons of the genera abundances formed a correlation network, where each node represents one genus and each edge stands for a strong and significant correlation between the nodes. The co-correlation network was visualized using CYTOSCAPE.

All statistical analyses were performed in the R environment (http://www.r-project.org) using the vegan^63^, igraph^64^ and gplots^65^ packages unless otherwise indicated.

## Electronic supplementary material

Supplementary Material

## Data Availability

The sequences produced in this study are available in the NCBI Sequence Read Archive (Bioproject PRJNA358858). The accession numbers of the bacteria and fungi in the different biosamples are SAMN06186591-SAMN06186600.
