# Author Correction: Bacterial and fungal gut communities of *Agrilus mali* at different developmental stages and fed different diets

**DOI:** 10.1038/s41598-019-41873-z

**Published:** 2019-05-23

**Authors:** Zhengqing Zhang, Shuo Jiao, Xiaohui Li, Menglou Li

**Affiliations:** 1 Laboratory of Forestry Pests Biological Control, College of Forestry, Northwest A&F University, Yangling, Shaanxi 712100 China; 2 College of Urban and Environmental Sciences, Peking University, Beijing, 100871 China

Correction to: *Scientific Reports* 10.1038/s41598-018-34127-x, published online 23 October 2018

This Article contains errors in Figure 1, where 4 microbial diversity patterns were omitted. The correct Fig. 1 appears below.

**Figure 1:**
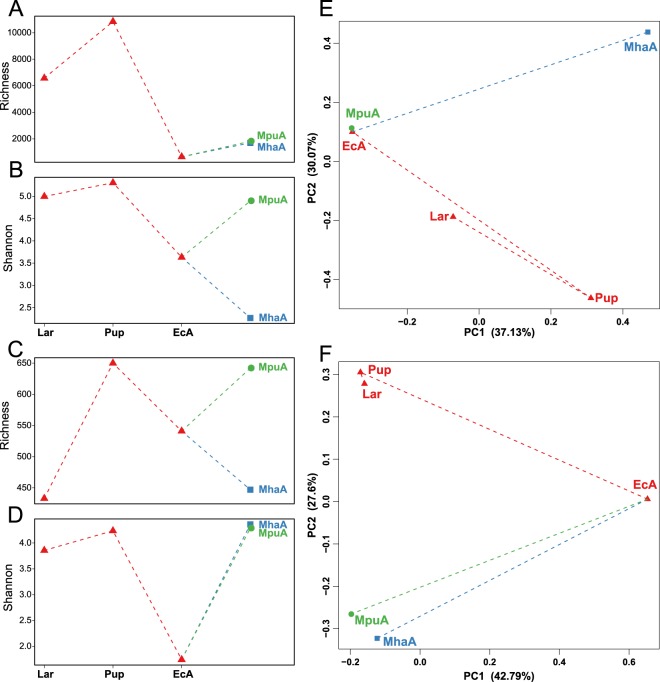
Microbial diversity patterns for *A. mali* gut microbiotas. Alpha-diversity in bacterial (**A,B**) and fungal (**C,D**) gut communities of *A. mali* gut. Beta-diversity in bacterial (**E**) and fungal (**F**) gut communities estimated via principal coordinate analysis (PCoA) based on Bray-Curtis distance.

As a result, the Figure legend,

“Microbial diversity patterns for *A. mali* gut microbiotas. Alpha-diversity in bacterial (**A,C**) and fungal (**B,D**) gut communities of *A. mali* gut. Beta-diversity in bacterial (**E**) and fungal (**F**) gut communities estimated via principal coordinate analysis (PCoA) based on Bray-Curtis distance.”

should read:

“Microbial diversity patterns for *A. mali* gut microbiotas. Alpha-diversity in bacterial (**A,B**) and fungal (**C,D**) gut communities of *A. mali* gut. Beta-diversity in bacterial (**E**) and fungal (**F**) gut communities estimated via principal coordinate analysis (PCoA) based on Bray-Curtis distance.”

