# Supplementary material for: Bacterial and fungal gut communities of Agrilus mali at different developmental stages and fed different diets
Source: Sci Rep. 2018 Oct 23;8:15634. doi: 10.1038/s41598-018-34127-x (PMC6199299; doi:10.1038/s41598-018-34127-x)

Supplementary materials for

Bacterial and fungal gut communities of *Agrilus mali* at different developmental stages and fed different diets

Zhengqing Zhang[a], Shuo Jiao[a,b], Xiaohui Li[a], Menglou Li[a*]

[a]: Laboratory of Forestry Pests Biological Control, College of Forestry, Northwest A&F University, Yangling Shaanxi 712100, China

[b]: College of Urban and Environmental Sciences, Peking University, Beijing 100871, China

*Corresponding author: Menglou Li

Email addresses: limenglou@hotmail.com

Tel: +86-029-87082125

Fax: +86-029-87082125

Table S1 The relative abundance and affiliation of first 15 bacterial core taxa.

OTU ID	Phylum	Class	Order	Family	Genus	Relative Abundance (%)
Otu00247	Proteobacteria	Gammaproteobacteria	Enterobacteriales	Enterobacteriaceae	*Klebsiella*	9.98
Otu00064	Proteobacteria	Gammaproteobacteria	Enterobacteriales	Enterobacteriaceae		5.80
Otu19549						3.79
Otu00986	Proteobacteria	Betaproteobacteria	Burkholderiales	Alcaligenaceae		2.92
Otu19539						2.60
Otu19502	Proteobacteria	Alphaproteobacteria	Rickettsiales			2.23
Otu19541						1.67
Otu19537						1.51
Otu01188	Proteobacteria	Gammaproteobacteria	Xanthomonadales	Xanthomonadaceae	*Stenotrophomonas*	1.16
Otu19674						0.97
Otu19681						0.78
Otu19561						0.69
Otu19576						0.50
Otu00252	Proteobacteria	Gammaproteobacteria	Enterobacteriales	Enterobacteriaceae	*Serratia*	0.46
Otu00571	Proteobacteria	Gammaproteobacteria	Pseudomonadales	Moraxellaceae	*Enhydrobacter*	0.44

Table S2 The relative abundance and affiliation of first 15 fungal core taxa.

OTU ID	Phylum	Class	Order	Family	Genus	Relative Abundance (%)
Otu0014	Ascomycota	Eurotiomycetes	Eurotiales	Trichocomaceae	*Aspergillus*	12.03
Otu0101	Basidiomycota	Wallemiomycetes	Wallemiales	Wallemiaceae	*Wallemia*	6.86
Otu1424						5.22
Otu0011	Ascomycota	Dothideomycetes	Pleosporales	Incertae_sedis	*Phoma*	4.71
Otu1426	Ascomycota	Saccharomycetes	Saccharomycetales	Incertae_sedis	*Candida*	4.25
Otu0015	Ascomycota	Eurotiomycetes	Eurotiales	Trichocomaceae	*Penicillium*	3.93
Otu1425	Basidiomycota	Tremellomycetes	Tremellales	Incertae_sedis	*Cryptococcus*	3.42
Otu0187	Ascomycota	Dothideomycetes	Incertae_sedis	Myxotrichaceae		2.14
Otu0001	Ascomycota	Eurotiomycetes	Eurotiales	Trichocomaceae	*Aspergillus*	2.05
Otu0044	Ascomycota	Dothideomycetes	Capnodiales	Davidiellaceae	*Cladosporium*	1.50
Otu1406	Ascomycota					1.44
Otu0066	Ascomycota	Sordariomycetes	Hypocreales	Nectriaceae	*Fusarium*	1.29
Otu0023	Ascomycota	Dothideomycetes	Pleosporales	Pleosporaceae	*Alternaria*	1.25
Otu0166	Ascomycota	Eurotiomycetes	Eurotiales	Trichocomaceae	*Aspergillus*	1.12
Otu1412	Ascomycota					1.05

Fig. S1 The rarefaction curves of Shannon-Wiener index for bacterial (A) and fungal (B) samples.

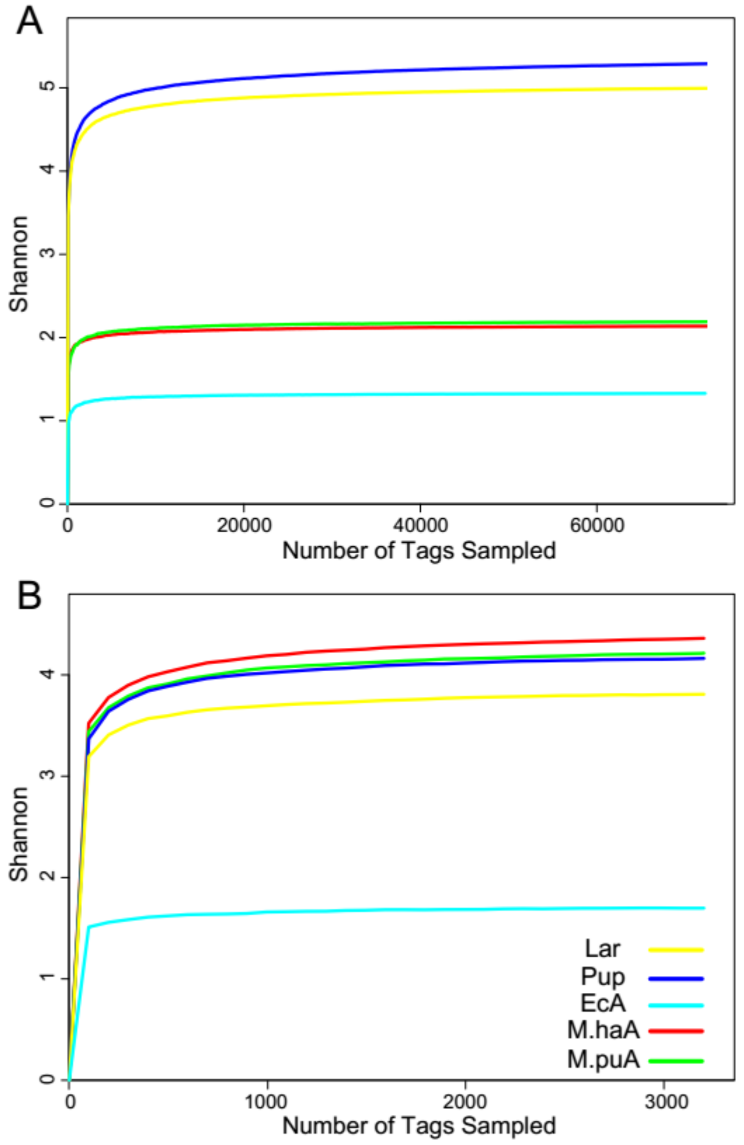

Fig. S2 The relative abundances of phyla in different gut microbiome of *A. mali*.

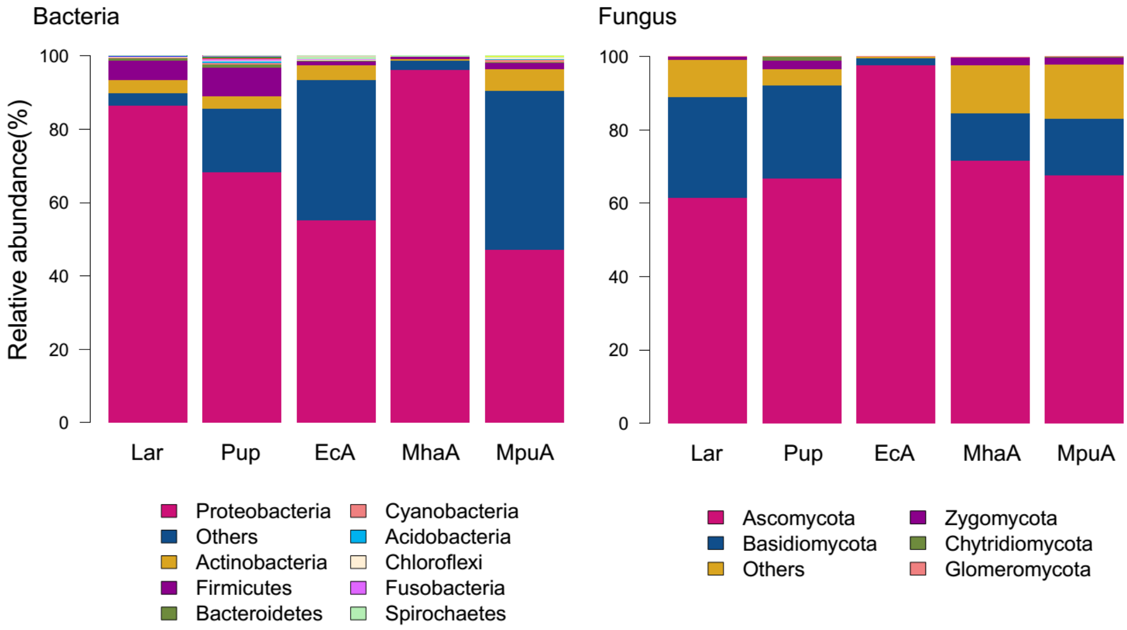

Fig. S3 The relative abundances of classes in different gut microbiome of *A. mali*.

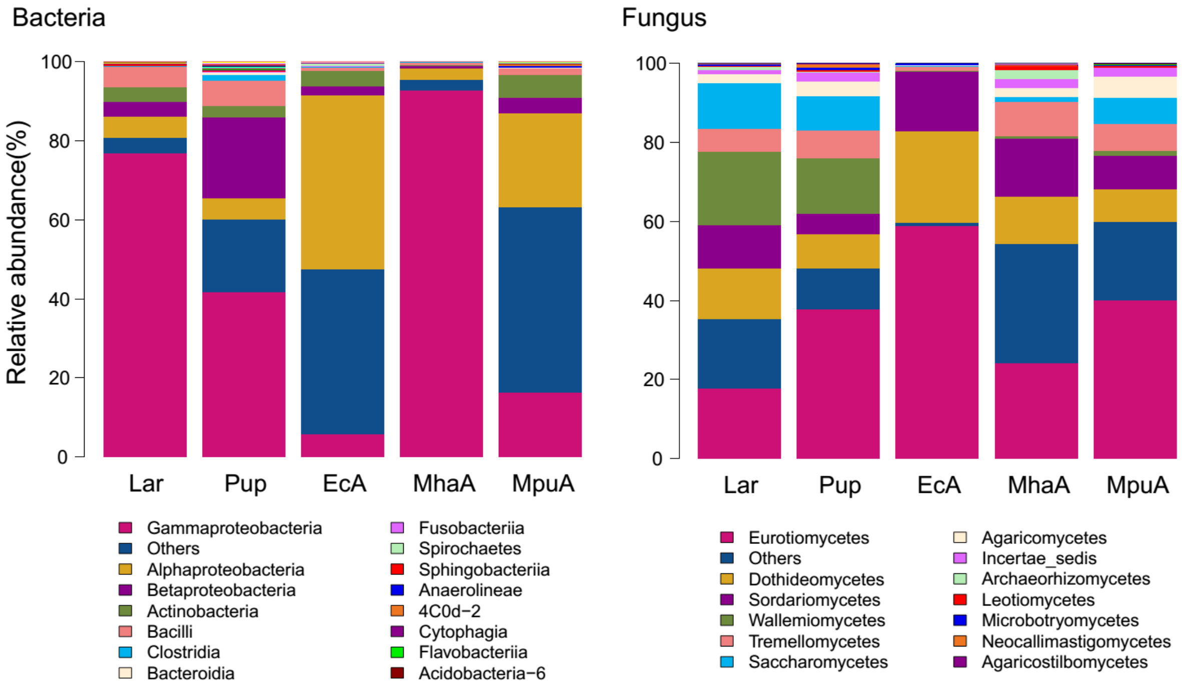

Fig. S4 The proportions of core taxa accounted for the reads in bacterial and fungal communities of different gut microbiome of *A. mali*.

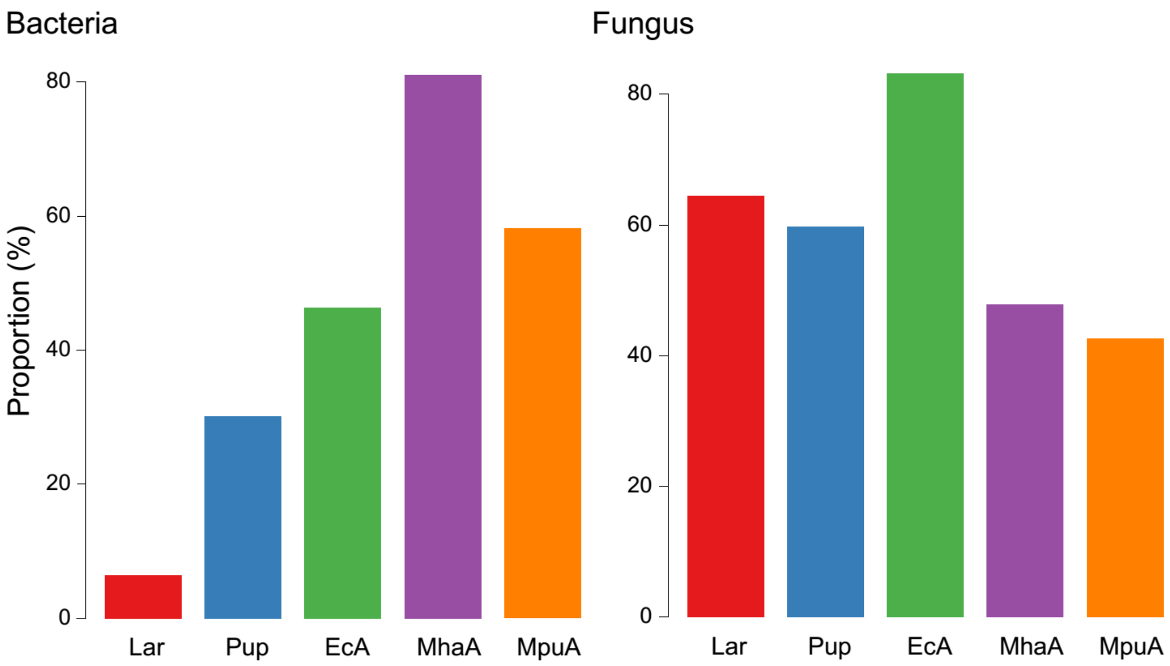

Supplement: Supplementary file 1 — Supplementary Material [file 41598_2018_34127_MOESM1_ESM.pdf]
